# UBXD Proteins: A Family of Proteins with Diverse Functions in Cancer

**DOI:** 10.3390/ijms17101724

**Published:** 2016-10-14

**Authors:** Khosrow Rezvani

**Affiliations:** Division of Basic Biomedical Sciences, Sanford School of Medicine, The University of South Dakota, 414 E. Clark Street, Lee Medical Building, Vermillion, SD 57069, USA; khosrow.rezvani@usd.edu; Tel.: +1-605-658-6383; Fax: +1-606-677-6381

**Keywords:** UBXD family, cell proliferation, apoptosis, tumor, animal models, xenografts, targeted therapy

## Abstract

The UBXD family is a diverse group of UBX (ubiquitin-regulatory X) domain-containing proteins in mammalian cells. Members of this family contain a UBX domain typically located at the carboxyl-terminal of the protein. In contrast to the UBX domain shared by all members of UBXD family, the amino-terminal domains are diverse and appear to carry out different roles in a subcellular localization-dependent manner. UBXD proteins are principally associated with the endoplasmic reticulum (ER), where they positively or negatively regulate the ER-associated degradation machinery (ERAD). The distinct protein interaction networks of UBXD proteins allow them to have specific functions independent of the ERAD pathway in a cell type- and tissue context-dependent manner. Recent reports have illustrated that a number of mammalian members of the UBXD family play critical roles in several proliferation and apoptosis pathways dysregulated in selected types of cancer. This review covers recent advances that elucidate the therapeutic potential of selected members of the UBXD family that can contribute to tumor growth.

## 1. Introduction

Ubiquitin regulatory X domain-containing proteins (the UBXD family) is a subclass of proteins with ubiquitin-related protein motifs [1]. In mammalian cells, the UBXD family has 13 members [2] divided into two main groups according to the arrangement of their ubiquitin-related protein motifs. Members of Group I (the UBA-UBX group) contain an ubiquitin-associated (UBA) domain located at the amino-terminus of the protein and a UBX domain located at the carboxyl-terminal of the protein. There are five members of this group: UBXD7 (UBXN7), UBXD8 (FAF2), UBXD10 (P47, NSFL1C), UBXD12 (FAF1), and UBXD13 (SAKS1, and UBXN1). Some members of Group I carry extra ubiquitin-related domains such as UIM (ubiquitin-interacting motif) and UBL (ubiquitin-like domain) [3]. Group II (the UBX group) has eight members (UBXD1 (UBXN6), UBXD2 (Erasin, UBXN4), UBXD3 (UBXN10), UBXD4 (UBXN2A), UBXD5 (Socius, COA-1, UBXN11), UBXD6 (Rep-8, UBXN8), UBXD9 (TUG, ASPL, ASPSCR1), and UBXD11 (P37, UBXN2B)) with a UBX domain as their only ubiquitin-related domain [3]. UBXD9 (TUG, ASPL) [4] is unlike the rest of the UBXD members in Group II as it has two UBL domains located at the amino-terminus of the protein in addition to its UBX domain [5,6]. The presence of diverse domains and their different combinations in the UBXD family allow these proteins to have different functional properties. These differences allow them to bind to a selected set of partners and cross-talk with different protein complexes in a subcellular localization-dependent manner [4,7,8,9,10,11,12,13,14,15,16]. Furthermore, the presence of other ubiquitin-related motifs besides the UBX domain enable UBXD proteins to exert non-redundant functions in the ubiquitin-proteasome pathway [17,18].

The UBX domain, with around 80-residues, has the same three-dimensional structure described for ubiquitin. However, UBX domains are unable to conjugate to other proteins or be a part of mixed UBX-ubiquitin chains. In contrast to the ubiquitin protein, a double glycine motif and suitably positioned lysine side-chains are absent in the structure of UBX domains [19]. The UBX domain enables all members of the UBXD family to bind to the multifunctional AAA-ATPase p97/VCP protein [20] via the amino terminal domain of p97 [7,21,22,23]. The hydrophobic pocket between the two subdomains of the p97 N-terminal domain is the binding site for the UBX domain. Protein-protein interaction studies indicate that p97 has more affinity for binding to the UBX domain than ubiquitin protein [21]. By binding to p97 associated with the endoplasmic reticulum (ER) lumen, UBXD proteins turn into a critical cofactor of the ER-associated degradation pathway, known as the ERAD pathway [13,24,25,26,27,28]. While the p97 complex is a key protein in the ERAD system, p97’s protein quality control function is additionally important for other subcellular organelles. For example, p97 is involved in autophagy and endosomal sorting pathways as well as protein degradation processes at the outer mitochondrial membrane and cytoplasmic compartment [29]. The multi-directional function of p97 has extended the roles of its main partner proteins, UBXD proteins, beyond the ERAD system, such as UBXD7, which functions in chromatin-associated processes in the presence of p97 [30].

A network proteomics study showed that proteins in the UBA-UBX group recognize K48 and K11-linked chains, preferably K11, and bind to different families of E3 ubiquitin ligases [3]. In particular, these results indicate that the UBA-UBX group plays a critical role in the regulation of p97 in the ubiquitin–proteasome system [15,17,31]. While the functions of some members of the UBX group (Group II) have been defined [24,28], the specific functions of different members of the UBX group in the ubiquitin-proteasome and p97 pathways remain obscure. In addition, their protein interaction networks, and their functions unrelated to the ubiquitin-proteasome pathway have not been well-studied.

**Figure 1:**
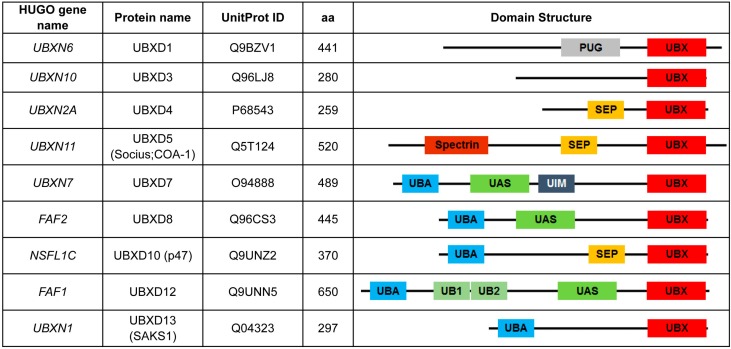
Schematic domain structures of the UBXD proteins. Linear representations of the domain structures of the nine members of the UBXD protein family involved in diverse tumorigenic pathways. The ubiquitin-regulatory X (UBX) domain is located at the C-terminus of proteins and is a defining feature of the family. Diverse domains and their different combinations located at the N-terminus of proteins enable UBXD proteins to bind to selected partners/substrates and contribute to diverse functions in different cell compartments. PUG (PNGase/UBA or UBX) domain, also known as PUB domain, binds to p97 protein. The SEP domain is named for Saccharomyces cerevisiae Shp1, Drosophila melanogaster eyes closed gene (eyc), and vertebrate p47. UAS and UIM stand for FAS-associated factor 1 and Ubiquitin-Interacting Motif, respectively. UB1 and UB2 are ubiquitin homologous domain 1 and 2, respectively.

Similar to the ERAD pathway, several recent reports have suggested positive and negative regulatory functions for UBXD proteins in various types of cancers. These UBXD proteins bind to certain proteins involved in proliferation and apoptosis pathways. In a p97/ubiquitin-dependent or p97/ubiquitin-independent manner, UBXD proteins modulate key control points in these two dysregulated pathways in cancer [32]. This study provides a systematic review of UBXD proteins and their functions in different types of cancer, covering both basic and clinical aspects. The current evidence validates the UBXD family as a druggable target with potential antitumor activity (Figure 1).

## 2. UBXN6, a Potential Predictor of Postoperative Recurrence in Gastric Cancer

Patients with advanced gastric cancer have a poor prognosis even after curative resection. Advanced gastric tumors are invasive and are responsible for 40% of deaths due to tumor recurrence [33]. The progression of gastric cancer is supported by multiple genetic events that lead to changes in the expression level of several genes. While clinicopathologic studies can assess the risk of recurrence, such an approach can be inadequate in individual patients. To find an alternative but complementary method of risk assessment, Motoori et al. conducted gene expression profiling with 2304 genes using 60 patients with advanced gastric cancer. Their high-throughput quantitative RT-PCR approach provided a list of 29 diagnostic genes. Follow-up analysis showed twelve genes were upregulated and seventeen genes were relatively downregulated in these patients. UBXN6 (Figure 1) was among the downregulated genes, with a *p*-value of 0.00764 [34]. More studies are needed to determine whether UBXD1 gene expression can be a reliable clinical predictor of recurrence in patients with advanced gastric cancer after curative resection.

## 3. UBXN10, Ciliogenesis, and Human Cancer Cells

Studies suggest that a defect in ciliogenesis can promote cancer development [35]. Dysregulation of primary cilia has been reported in several human cancers, including breast cancer [36], renal cell carcinoma [37], and pancreatic cancer [38]. In addition, the loss of cilia and elevation of tumor incidence in basal cell carcinoma and medulloblastoma have been observed in mouse models [38]. While the functions of primary cilia have been highlighted in cancer initiation, progression, and targeted drug efficacy [38], more studies are needed to unveil the functions and molecular mechanisms of ciliogenesis in tumorigenesis.

A recent systematic proteomic analysis of UBXD proteins in human cells revealed that UBXN10 (Figure 1) binds to the intraflagellar transport B (IFT-B) complex, a critical protein in cilia biogenesis [39]. UBXN10 needs to bind p97 to localize within cilia, and loss of UBXN10 and p97 leads to a significant decrease in the number of cilia [40]. Further characterization of UBXN10’s functions in ciliogenesis may clarify the importance of UBXN10 in tumorigenesis initiated by dysregulated ciliogenesis.

## 4. UBXN2A, a Potential Target for Cancer Therapy

UBXN2A, with 259 amino acids, has a SEP (Shp1, Eyc, and P47) domain located at the N-terminus and a UBX domain located at the C-terminus of the protein (Figure 1). UBXN2A was first reported as a regulatory protein involved in the protein trafficking of nicotinic receptors in the central nervous system [27]. Similar to other members of the UBXD family [31], UBXN2A binds to p97 and regulates protein quality and the proteasomal degradation of proteins maintained by the ERAD pathway. UBXN2A binds to several E3 ubiquitin ligases, including CHIP (carboxyl terminus of Hsc70 interacting protein) ubiquitin E3 ligase [28]. Binding to different E3 ubiquitin ligases enables UBXN2A to regulate the stability of several diverse substrates in a cell- and tissue-dependent manner [28,41].

A proteomic approach for identifying cellular proteins interacting with UBXN2A in a HCT116 colon cancer cell line revealed UBXN2A binds to mortalin-2 (GRP75, HSPA9, mot-2) protein. Mot-2 is a subclass of heat shock protein 70 (HSP70) with a typical ATPase domain at the N-terminus and a substrate-binding domain (SBD domain) at the C-terminus of the protein [42]. A mitochondrial localization signal guides mot-2 into the mitochondrial matrix, where mot-2 imports and folds cytoplasmic proteins entering into mitochondria [42]. However, mislocalization of mot-2 in the cytoplasmic and nuclear compartments acquire different binding partners [43] and turn mot-2 into a dominant oncoprotein promoting tumorigenesis. A set of fractionation experiments combined with high-resolution single-cell image analysis showed that mot-2 is significantly upregulated in the cytoplasm and nuclear compartments of several human cancer cell lines in comparison to non-cancerous cell lines. Immunostaining of mot-2 in human normal and cancer ovarian tissues revealed mot-2 enrichment in the nuclei of cancer cells but absence from the nuclear compartment in normal ovarian tissue [44,45,46]. A similar upregulation and sub-cellular distribution of mortalin has also been reported in human liver cancers and melanoma samples [47,48,49]. Certainly, further immunohistochemical experiments are needed to understand the abnormal localization of mot-2 in different human tumors. In the cytoplasm, one of the main targets of mot-2 is p53 tumor suppressor protein. By binding to p53, mot-2 sequesters p53 in the cytoplasm, eliminating p53’s transcriptional activities [50,51]. Convincing evidence indicates the inhibition of mot-2 protein in cancer cells can have a potential clinical benefit in several types of cancers, including colorectal and breast cancers [43,47,52,53,54].

**Figure 2:**
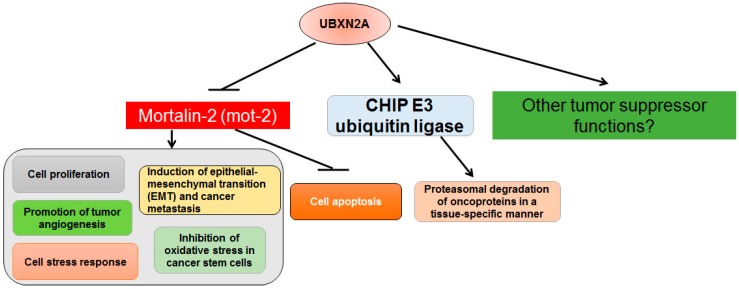
Overview of the UBXN2A binding partners which can mediate its tumor suppressor abilities. The presence of UBXN2A can inhibit mortalin-2 oncoprotein and prevent tumor formation by inducing apoptosis and/or and inhibiting cell growth, adhesion, and migration. Furthermore, UBXN2A can regulate the proteasomal degradation of CHIP’s substrates, including oncoproteins in cancer cells. Further studies will provide a better picture of the anti-cancer functions of UBXN2A and its potential therapeutic benefits.

The SEP domain of UBXN2A attaches to the protein binding pocket located within the SBD domain of mot-2 [41]. By binding to mot-2, UBXN2A unsequesters p53 tumor suppressor proteins, which leads to activation of the apoptotic pathway downstream of p53 [55]. UBXN2A is dominantly localized in the nucleus in colon cancer cell lines and translocates to cytoplasmic compartments upon genotoxic stress, where it binds to mot-2 protein [16]. Induction of UBXN2A induces cell apoptosis and reverses tumor progression in both in vitro and xenograft mouse models [56]. In addition, overexpression or silencing of UBXN2A increases response to conventional chemotherapy in cancer cells with enriched mot-2 [41]. Induction of UBXN2A shows minimal effect on normal cells because the large portion of mot-2 is located in mitochondria [55,56]. Together, the above evidence indicates that UBXN2A can act as a potential target therapy in cancer cells with a high level of mot-2 (Figure 2).

As previously highlighted, mot-2 and its protein networks allow this oncoprotein to be involved in several tumorigenic pathways in a tissue-dependent manner [43]. Further studies are required to dissect the various mechanisms underlying UBXN2A anti-cancer functions upon its binding to mortalin and how UBXN2A-dependent inhibition of the mortalin oncoprotein pathway can alter the apoptotic efficacy of chemotherapy.

## 5. UBXN11, an Antigen Recognized by Colon Tumor-Reactive T Cells

The identification of antigens processed and presented at the surface of tumor cells plays a critical role in cancer immunotherapy. These antigens can be identified by T cells that were initially generated after tumor stimulation. However, a limited number of target molecules have been found by colon tumor-reactive T cells, which has limited the development of immunotherapies for patients with colorectal cancer. A study conducted by Maccalli et al. revealed UBXN11 (Figure 1) (UBXD5, Socius or colorectal tumor-associated antigen-1 (COA-1)) is a potential candidate recognized by CD4^+^ T lymphocytes in colorectal and melanoma tumors [57].

## 6. UBXD7, a Selective Modulator of HIF-1α

An altered protein expression profile in response to a hypoxic microenvironment enables hypoxic tumors to become more aggressive and resistant to anti-cancer treatments. Hypoxia inducible factor 1α (HIF-1α), as a transcription factor, plays a major role in tumors following hypoxia, promoting tumor aggressiveness and invasiveness as well as potentially compromising responses to radiotherapy and chemotherapy [58]. Inhibition of HIF-1α leads to disruption of multiple pathways, including cell survival, glucose metabolism, cell invasion, and angiogenesis. Therefore, HIF-1α inhibition is a promising and effective strategy for the treatment of cancers [59]. 

A study conducted in Deshaies’s group showed that the UBXD7 protein targets HIF-1α for ubiquitination and degradation via the p97 complex. UBXD7 belongs to the UBA-UBX group with a UBA domain located at the N-terminus, a UBX domain at the C-terminus, and a UAS and UIM domain in the middle of its protein structure (Figure 1). A major part of endogenous UBXD7 is not associated with p97 in cells, and they are in an inactive state due to intra- or intermolecular interaction of the UBA and UBX domains. Upon binding of the UBA domain of UBXD7 to ubiquitinated HIF-1α, UBXD7 can actively facilitate p97’s interaction with CUL2/VHL E3 ubiquitin ligase and HIF-1α. Formation of p97-UBXD7-HIF-1α-CUL2/VHL E3 ubiquitin ligase complex initiates proteasomal degradation of HIF-1α. The degradation pattern of HIF-1α in the presence of silenced p97 or UBXD7 suggests that the UBXD7-p97 complex targets only a subset of HIF-1α (probably in a particular assembly state, i.e., bound to HIF-1β). The UBXD7-dependent degradation of HIF-1α is a slower degradation pathway than the UBXD7-independent degradation of HIF-1α provided by other proteasome-targeting factors [3]. Following the initial discovery by Deshaies’s group, another study showed that UBXD7 can directly bind to neddylated CUL2. By binding to the neddylated form of CUL2, UBXD7 uses its UBA and UBX domains to recruit ubiquitinated HIF-1α and p97 complex, respectively. However, overexpression of UBXD7 indicates that the docking mechanism of UBXD7 has a negative regulatory effect on the ubiquitin-ligase activity of CUL2 and leads to accumulation of HIF-1α. On the other hand, nuclear localization of UBXD7 strongly suggests that UBXD7/p97 specifically targets nuclear HIF-1α [8]. These two studies show the complexity of UBXD7’s regulatory roles downstream and upstream of the ubiquitin-proteasome pathway. Further studies are needed to understand the regulatory effect of UBXD7 on HIF1α’s stability and functions in the presence and the absence of hypoxia in cancer cells. Of particular interest is whether targeting UBXD7 will have a therapeutic benefit on hypoxic tumors.

## 7. UBXD8, an Inhibitor of Neurofibromin

Neurofibromin functions as a tumor suppressor protein by inhibiting the Ras-mediated signaling pathway. Loss of neurofibromin leads to formation of neurofibroma, a benign tumor arising from the peripheral nerve in Schwann cells [60]. Phan et al. reported that neurofibromin stability is regulated by UBXD8 (ETEA, FAF2-Figure 1) [61]. More interestingly, silencing UBXD8 protein reduces Ras signal activity and downregulates the ERK and AKT pathways. Therefore, inhibition of UBXD8 could be a potential therapeutic target in patients with neurofibroma [61].

## 8. P47, a Negative Regulator of the NF-κB Pathway in Cancer Cells

The p47 (UBXN2C, UBXD10, NSFL1C) protein has a UBA [62], a SEP [63,64], and a UBX domain located in the N-terminus (1–45 aa), the center (179–246 aa), and the C-terminus (286–370 aa) of p47 protein, respectively (Figure 1). Ubiquitin-binding motifs allow p47 to bind to the p97 complex and regulate p97-dependent degradation of ER substrates as part of the ERAD pathway [65]. The association of the p47 and p97 complex is essential during reassembly of the Golgi and ER at the end of mitosis [66].

The UBA domain of p47 binds to mono-ubiquitin conjugate and is required for the function of p47 in mitotic Golgi reassembly [67]. Localization of p47 in the nucleus during interphase and its phosphorylation on Serine-140 by Cdc2 at mitosis plays a critical role in p47 during Golgi disassembly-assembly [68].

In addition to Golgi reassembly, p47 is crucial for the p97-mediated membrane fusion events [69] that occur during assembly of the smooth ER domain of the transitional endoplasmic reticulum (tER) [70] as well as the nuclear envelope where the p97–p47 complex is necessary for nuclear envelope (NE) growth followed by NE formation [71].

The precise function of the SEP domain is still under examination. This is an eukaryotic domain, occurring frequently and mainly in single units and commonly associated closely with a UBX domain [72]. A set of nuclear magnetic resonance (NMR) studies suggested that the SEP domain of p47 can function as a reversible competitive inhibitor of the lysosomal cysteine protease cathepsin L [64]. Cathepsin L is considerably overexpressed in a wide variety of cancers [73] and promotes tumor growth, migration, invasion, angiogenesis, and metastasis [74]. It has been illustrated that cathepsin L can be an independent and reliable prognostic marker in human breast cancer [75,76]. In addition, pharmacological targeting of cathepsin L has proven to be of viable therapeutic benefit for the treatment of cancer [77]. Therefore, inhibition of cathepsin L by the SEP domain can be considered a promising target for cancer therapy. The SEP domain along the UBX domain is also responsible for trimerization of p47, which is essential to make a highly stable complex with one hexamer of p97 [63,78].

The nuclear factor-κB (NF-κB) pathway is among the common signaling pathways in pre-neoplastic and malignant cells and frequently is deregulated in cancer [79]. Activation of this pathway induces downstream genes that are critical for tumor progression, tumor metastasis, and drug resistance [80]. NF-κB’s target genes are involved in cell proliferation, apoptosis, and angiogenesis as well as the promotion of invasion/metastasis and senescence in cancer cells [81]. It has been well accepted that solid tumors such as prostate, colorectal, breast, and liver cancers as well as hematologic cancers such as leukemia utilize a continued activation of NF-κB to progress and escape cell death [81,82,83,84]. The dominant tumorigenic role of NF-κB in cancer has turned this pathway into a potential molecular target for cancer therapy [85,86].

The activation of the NF-κB pathway starts by means of specific stimulators that trigger phosphorylation and proteasome-mediated degradation of the inhibitor of kappa B (IκB). Degradation of IκB leads to unsequestration of NF-κB subunits (p65/RelA and p50/NF-κB1) in the cytoplasm followed by their translocation into the nucleus where the NF-κB dimers (p65 and p50) induce a large number of tumorigenic genes [87]. A network of signaling intermediates and their associated proteins are necessary for NF-κB signaling cascade. One of these proteins is nuclear factor-κB (NF-κB) essential modulator (NEMO). NEMO is a component of the IκB kinase (IKK) complex and mediates NF-κB signaling by binding to K63 ubiquitin chains [88]. Point mutation experiments and pharmacological inhibition of NEMO illustrate the critical role of NEMO in IKK activation [89,90].

**Figure 3:**
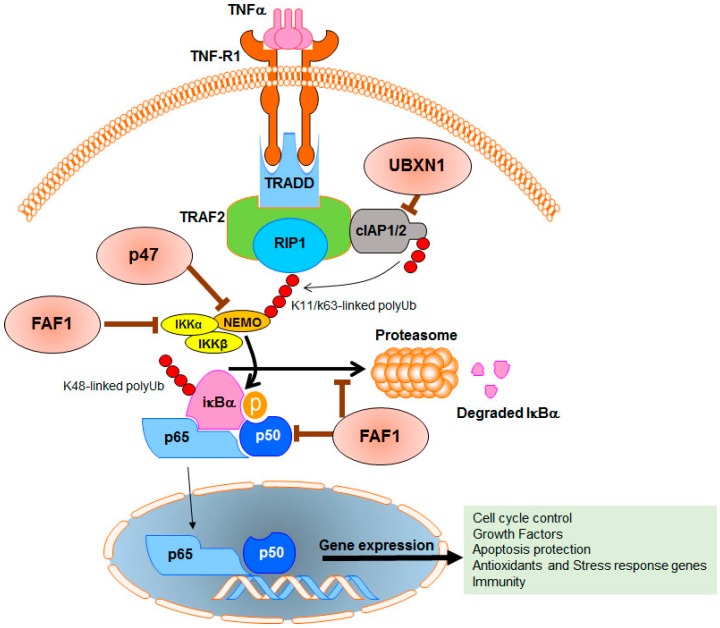
Functions of three members of the UBXD protein family (UBXN1, P47, and FAF1) that are potentially involved in the prevention of cancer. It has been well established that aberrant regulation of nuclear factor-kappaB (NF-κB) and its downstream cascade are involved in several aspects of tumor progression as well as resistance to chemo- and radio-therapies. The NF-κB signaling pathway is regulated by a network of intermediate and adaptor proteins. UBXN1, p47, and FAF1 can target and inhibit several of these key regulatory proteins and block transcription of oncogenic genes activated by the NF-κB pathway. TNFα = tumor necrosis factor α, TNF-R1 = tumor necrosis factor receptor 1, TRADD = TNFR1-associated death domain protein. TRAF2 = tumor necrosis factor receptor-associated factor 2, NEMO = NF-κB essential modulator, IKKα and IKKβ = IκB kinase complex (IKK)-α and IκB kinase complex (IKK)-β, cIAP1 and cIAP2 = cellular inhibitor of apoptosis 1 and 2. The thin black arrow stands for the poly ubiquitination process. Initiation or activation is shown by thick black arrows and inhibition is shown by brown T-bars.

Shibata et al. reported that the p47 protein can function as an inhibitor in the NF-κB pathway. It was found that p47 binds to ubiquitinated NEMO, preferably via lysin63-linked and linear polyubiquitination chains versus lys48-linked polyubiquitin chains associated with NEMO. P47 uses the UBA domain to bind ubiquitinated NEMO. By binding to NEMO, p47 causes the lysosomal-dependent degradation of polyubiquitinated NEMO, resulting in the prevention of IKK activation and, consequently, suppression of the expression of the target genes of NF-κB. Suppression of NF-κB’s target genes reduces the level of several key anti-apoptotic proteins such as cellular FLICE (FADD-like IL-1β-converting enzyme)-inhibitory protein (c-FLIP) [91] and the inhibitor of apoptosis (IAP) [92] protein, followed by activation of several apoptotic proteins, including caspase proteins. In addition, truncated forms of p47 revealed that the SEP domain can effect p47/NEMO interaction and is also involved in IKK inhibition. These findings suggest that p47 plays an anti-cancer function in cancer cells by interfering with the NF-κB pathway. This is accomplished by reversing this tumorigenic pathway in cancer cells via degradation of NEMO independent of the ubiquitin-proteasome pathway (Figure 3) [93].

Expectedly, the Gene Expression Omnibus database and the Oncomine database indicate that the mRNA and protein levels of p47 are reduced in different types of human solid tumors such as breast carcinoma and prostate cancer. In addition, p47 expression is dramatically reduced at both the mRNA and protein levels in patients with T cell leukemia and human T cell leukemia virus type 1-infected cell lines in which the NF-κB pathway is constitutively active [93]. Therefore, enhanced expression of p47 and its distinct inhibitory mechanism in the NF-κB pathway can be an effective therapy in tumors with a low level of p47 and a dysregulated NF-κB pathway.

## 9. FAF1, a Powerful Anti-Cancer Protein

FAS-associated factor 1 (FAF1) (UBXN3A), is a UBX domain-containing protein (Figure 1) that has several important roles in normal cells, including development and neuronal cell survival [94]. On the other hand, FAF1’s interaction with polyubiquitinated proteins, the p97 complex, heat shock protein 70 (HSP70), and the members of the Fas-signaling pathway enable this UBX domain-containing protein to play a critical role in the pathogenesis of human cancers [95]. Similar to other members of the UBXD family, FAF1 functions as a cofactor of p97 in the ERAD pathway [12,96]. Besides a UBX domain, FAF1 has several ubiquitin-like domains, including two ubiquitin homologous domains (UB1 and UB2) as well as an ubiquitin-associated domain (UAS), which enable FAF1 to regulate proteasomal degradation of selective substrates in cooperation with the p97 complex. In addition, FAF1 has an UBA domain at its N-terminus that can bind to selected multi-ubiquitinated proteins and promote their degradation as part of the ERAD pathway [12,97]. Interestingly, overexpression of hFAF1 interferes with the proteasomal degradation of IκBα and leads to accumulation of total ubiquitinated proteins [98]. FAF1-dependent stabilization of IκBα which functions as a potential tumor suppressor factor [99,100,101] and accumulation of ubiquitinated proteins [102] explain the FAF1-dependent induction of apoptosis observed by Song et al. [98].

While ubiquitin-like domains are critical for the anti-cancer roles of FAF1, the presence of non-related ubiquitin domains further promotes the anti-cancer role of FAF1. The significant anti-cancer functions of FAF1 were further verified when studies reported the down-regulation of FAF1 in tumors [103]. FID (Fas-interacting domain) and a death effector domain interacting domain (DEDID) are two domains of FAF1 located at amino acids 1–181 and 181–381, respectively [95]. By binding to Fas protein, FAF1 potentiates Fas-induced apoptosis, demonstrating FAF1’s essential role in the regulation of apoptosis [104]. Besides binding to Fas protein, FAF1 binds to caspase-8 and FADD in both in vitro and in vivo models. FAF1 uses DEDID to bind the death effector domains (DEDs) of caspase-8 and FADD. FAF1-dependent apoptotic death is significantly decreased in both FADD- and caspase-8-deficient cells, indicating FAF1 facilitates activation of an extrinsic apoptosis pathway [105].

In another set of studies, it has been shown that a phosphorylated form of FAF1 can mediate the ubiquitin-independent, proteasome-dependent degradation of Aurora-A (Aur-A). Overexpression of FAF1 blocks Aur-A-induced centrosome amplification and leads to accumulated cells in the G2/M phase. Combining this later finding with FAF1’s roles in apoptosis suggests an important molecular cross-talk between constituents of the cell cycle and cell death machinery following FAF1 overexpression [106].

In another set of studies, Kinoshita et al. showed that FAF1 binds to PYRIN-containing apoptotic protease-activating factor 1-like proteins (PYPAFs, also called NALPs) via its FID domain. This protein-protein interaction allows FAF1 to negatively regulate the NF-κB signaling pathway that involves PYPAF1 [107]. 

In addition to FAF1’s functions as a component of the Fas death-inducing signaling complex explained earlier, FAF1 physically binds to NF-κB p65 via its DEDID to prevent translocation of NF-κB p65 into the nucleus, resulting in suppression of the NF-κB pathway upon tumor necrosis factor-α (TNF-α) stimulation [108]. Finally, FAF1 is able to bind to the leucine-zipper domain of IKKβ and interrupt the IKK complex assembly, resulting in suppression of IKK activation and its downstream signaling [109,110]. FAF1’s multiple-binding partners in NF-κB pathway allow FAF1 to effectively prevent inadvertent activation of the NF-κB pathway. The negative effect of FAF1 on the NF-κB pathway is a promising therapeutic strategy in human cancers with low FAF1 levels, such as cervical and gastric carcinomas, mantle cell lymphomas, and malignant mesothelioma, which show hyperactive NF-κB activity (Figure 3) [111,112].

Interaction of FAF1 with HSP70, a well-known anti-apoptotic protein and a poor prognostic factor [43], is another anti-cancer mechanism of FAF1. By binding to HSP70, FAF1 decreases the Hsp70 chaperone activity, accelerates heat shock-induced SAPK/JNK activation, and promotes proteasomal degradation of HSP70. FAF-1-dependent degradation of HSP70 reduces colony formation and induces apoptosis in cancer cells [97,103,113,114]. While FAF1 expression is low in ovarian cancer, the expression of HSP70 shows a significant increase in ovarian cancer in comparison to that in a normal ovary [103]. The opposite correlation between the expression of FAF1 and HSP70 in ovarian cancer highlights the prognostic and therapeutic potential of FAF1 in patients with ovarian cancer.

It has been shown that some tumor cells express the nonselective cation channel Vanilloid receptor-1 (VR1/TRPV1) TRPV1 which has a preference for Ca^2+^. Activation of TRPV1 by its ligand, capsaicin (CP), leads to apoptosis in cancer cells [115]. FAF1 can be associated with TRPV1 receptors [116]. It has been shown that incubation of murine tumor cells (methylcholanthrene-induced fibrosarcoma (Meth A) and CMS-5) with CP agonist reduces the cellular level of the TRPV1 associated protein FAF1. In a controversial study, the silencing of FAF1 in these two murine cell lines promoted apoptosis following treatment with CP [117]. These results are in contrast to the apoptotic effect of FAF1 seen in human cancer cell lines. This study was conducted in murine-derived tumor cells in the presence of CP, and Ghosh et al. observed no induction of apoptosis when they silenced FAF1 protein in the absence of CP in a CMS-5 fibrosarcoma cell line. The authors highlighted that their study needs further experimentation [117].

The above evidence indicates that FAF1 cross talks with diverse oncoprotein and tumor suppressor proteins. As a result, the loss of FAF1 anti-cancer functions may contribute to diverse hallmarks of cancer. Future studies will enable illustration of the therapeutic potential of FAF1 in various types of cancer and help improve understanding of the anti-cancer mechanism of the UBXD family in human cancers.

## 10. UBXN1, an Inhibitor of Cellular Inhibitors of Apoptosis Proteins (cIAP)

cIAP proteins are upregulated in several types of human cancer and are associated with tumor progression, poor prognosis, and chemoresistance. It has been well characterized that overexpressed cIAP proteins inhibit caspase activation and apoptosis triggered during the intrinsic and extrinsic apoptosis pathways [118]. In addition to inhibition of apoptosis, cIAP proteins have an important regulatory role in both canonical and non-canonical NF-κB signaling pathways [119]. Overall, the E3 ubiquitin ligase activity and its protein partners allow cIAP proteins to facilitate multiple aspects of tumor progression, including cell proliferation, mitosis, cell invasion, and metastasis [120,121]. Targeting the inhibitor of apoptosis proteins (IAPs) has become an attractive target for a new generation of targeted therapy in several types of cancer [122].

UBXN1 (SAKS1) is a UBX domain-containing protein that contains both UBX and UBA domains (Figure 1). A siRNA screen of UBA domain-containing proteins revealed that silencing three members of the UBXD family, UBXN1, p47, and FAF1, can markedly potentiate TNFα-triggered NF-κB activation [123]. The negative regulatory functions of FAF1 and p47 in the NF-κB pathway were previously covered in an earlier part of this review [93,108]. However, the overexpression experiments showed that UBXN1 has the strongest inhibiting ability on TNFα-triggered NF-κB activity in comparison to p47 and FAF1 [123]. Further experiments in cancer as well as immortalized noncancerous cell lines showed that knockdown of UBXN1 potentiates TNFα-triggered NF-κB signaling, suggesting UBXN1 is a physiological suppressor of the NF-κB pathway [123]. Overexpression of wild-type and truncated forms of UBXN1 showed that 1) UBXN1 acts as a negative regulator upstream of the TAK1 and IKK complex in the NF-κB signaling pathway, and 2) the presence of the UBA domain is necessary for UBXN1-mediated NF-κB inhibition [123].

Lys-63 ubiquitination of adaptor protein receptor-interacting protein 1 (RIP1) is a key step in the cascade of canonical NF-κB signaling pathway [124] mediated through tumor necrosis factor (TNF) receptor-1 (TNFR1) signaling [125]. As previously described, the E3 ubiquitin ligase activities of cIAP1 or cIAP2 are a crucial step for RIP1 polyubiquitination during NF-κB activation [119]. Binding cIAP1 and cIAP2 to the TNFR-associated death domain (TRADD)/TNFR-associated factor 2 (TRAF2) protein complex leads to Lys-63 ubiquitination of RIP1, which is necessary for downstream TAK1 and IKK complex recruitment [126]. A set of gain- and loss-of-function approaches confirmed that UBXN1 binds to cIAP1 protein and sequesters cIAP1 from recruiting to the TNFR1 signaling complex [123]. Reduction of cIAP-interacting TRAF2 decreases the ubiquitinated form of RIP1 within the TRAF2 complex, resulting in the inhibition of NF-κB signaling activity stimulated by TNFα. UBXN-1 sequestration of cIAP1 is specific, since p47 and FAF1 failed to impair cIAP1 recruitment to TNFR1 in response to TNFα. Interestingly, the absence of p97 had no effect on UBXN1-mediated NF-κB inhibition (Figure 3) [123].

In addition to cIAP1, UBXN1 uses its UBA domain to target and bind to K6-linked polyubiquitin chains conjugated to BRCA1. It has been well characterized that BRCA1 functions as a tumor suppressor for many cases of hereditary breast and ovarian cancer. At the same time, the UBX domain of UBXN1 binds to the BRCA1/BARD1 heterodimer. By binding to the BRCA/BARD1 heterodimer, UBXN1 significantly decreases the E3 ligase activity of BRCA1/BARD1. BRCA1 participates in tumor suppression and its ubiquitin ligase activity has a significant role in this tumor suppression [127]. Therefore, UBXN1 may facilitate tumor progression by negative regulation of BRCA1 [128]. UBXN1-dependent inhibition of pro-survival protein cIAP1 and tumor suppressor protein BRCA1 suggests a controversial role for UBXN1 cancer. Further studies will be required to clarify these opposing functions. One explanation for this controversial picture of UBXN1 function can be related to the subcellular localization of UBXN1 as it has been described for p27 protein [129,130].

## 11. Conclusions and Future Directions

**Figure 4:**
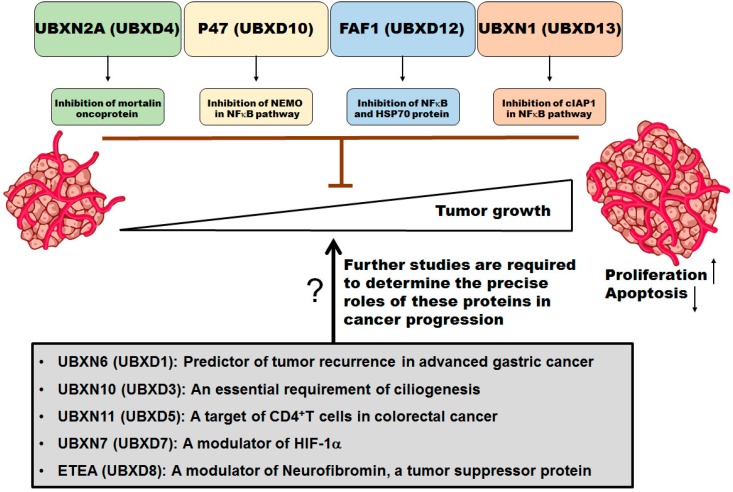
The UBXD family and cancer. UBXD family proteins are involved in a broad range of biological processes in normal and disease states, including cancers. Based on current literature, several members of the UBXD family contribute to oncogenic processes such as cell proliferation and apoptosis. A greater understanding of the interplay among UBXD family members, other binding partners, and regulators of tumorigenic pathways, particularly in animal models, is crucial for the design of a new generation of anti-cancer drugs targeting members of the UBXD family. Identification of the tumor-suppressing functions of UBXD proteins is important within a particular tissue or cell type. Targeting these novel tumor suppressor proteins can enhance the effectiveness of current treatment strategies without damaging normal cells. Initiation or activation is shown by arrows and inhibition is shown by brown T-bars. “?” = Further studies are needed.

The critical roles of the ubiquitin-proteasome system (UPS) in cell transformation and cancer progression have been well established [131,132,133]. Targeting the ubiquitin-proteasome pathway (UPS) with proteasome inhibitors in solid tumors has demonstrated a lack of clinical benefit [134] in contrast to its therapeutic benefit in hematological malignancies [135]. In addition, the primary generation of proteasome inhibitors can lead to severe side effects ranging from nerve damage and low blood counts to nausea and constipation [136] as well as drug resistance [137]. Even new generation proteasome inhibitors such as carfilzomib still have some serious limitations [138,139,140]. The marginal efficacy of proteasome inhibitors in solid tumors has encouraged scientists to start examining other components of the ubiquitin-proteasome pathways with higher specificity to cancer cells. Inhibition of deubiquitinating enzyme and the p97 complex are two alternative approaches that can modulate several key proteins that support oncogenic transformation and progression while avoiding unwanted effects on other cellular proteins, as observed with general proteasome inhibitors [141,142]. The evidence provided in this review has highlighted the regulatory functions of some members of the UBXD family in different types of cancers (Figure 4). Modulation of these ubiquitin-like proteins can affect specific pathways relied upon by cancer cells for accelerated and unregulated growth while leaving normal cells intact. Pre-clinical studies are the next step to elucidating whether the UBXD family is a potential target for the discovery of the next generation of non-genotoxic targeted therapies in human cancers.

This review covered only nine members of the UBXD family based on available data in the current literature. Future in vitro and in vivo studies will reveal whether the rest of UBXD family (UBXD2, UBXD6, UBXD9, and UBXD11) contributes to tumor formation and development. While current evidence strongly suggests that members of UBXD family have important roles in different types of cancer, the majority of studies and conclusions related to the function of UBXD family in cancers are based on in vitro experiments. Further in vivo studies in appropriate animal models and primary cell lines are needed to confirm the role of UBXD family member in more physiological conditions. Determination of RNA and protein levels of members of UBXD family in normal and diseased human tissues is essential information. This can further help define the specific functions of these proteins in a tissue- and disease-dependent manner. Optimization of reliable functional assays for individual members of the UBXD family is requisite for pre-clinical and clinical screening of these important proteins in different types of cancer.
